# The Use of Functional Electrical Stimulation on the Upper Limb and Interscapular Muscles of Patients with Stroke for the Improvement of Reaching Movements: A Feasibility Study

**DOI:** 10.3389/fneur.2017.00186

**Published:** 2017-05-10

**Authors:** Alicia Cuesta-Gómez, Francisco Molina-Rueda, Maria Carratala-Tejada, Eukene Imatz-Ojanguren, Diego Torricelli, Juan Carlos Miangolarra-Page

**Affiliations:** ^1^Motion Analysis, Ergonomics, Biomechanics and Motor Control Laboratory (LAMBECOM), Department of Physical Therapy, Occupational Therapy, Rehabilitation and Physical Medicine, Rey Juan Carlos University, Alcorcón, Spain; ^2^Tecnalia, San Sebastián, Spain; ^3^Spanish National Research Council (CSIC), Madrid, Spain; ^4^Chair of Rehabilitation and Physical Medicine, Fuenlabrada University Hospital, Madrid, Spain

**Keywords:** electric stimulation therapy, movement disorders, paresis, range of motion, stroke, upper extremity

## Abstract

**Introduction:**

Reaching movements in stroke patients are characterized by decreased amplitudes at the shoulder and elbow joints and greater displacements of the trunk, compared to healthy subjects. The importance of an appropriate and specific contraction of the interscapular and upper limb (UL) muscles is crucial to achieving proper reaching movements. Functional electrical stimulation (FES) is used to activate the paretic muscles using short-duration electrical pulses.

**Objective:**

To evaluate whether the application of FES in the UL and interscapular muscles of stroke patients with motor impairments of the UL modifies patients’ reaching patterns, measured using instrumental movement analysis systems.

**Design:**

A cross-sectional study was carried out.

**Setting:**

The VICON Motion System^®^ was used to conduct motion analysis.

**Participants:**

Twenty-one patients with chronic stroke.

**Intervention:**

The Compex^®^ electric stimulator was used to provide muscle stimulation during two conditions: a placebo condition and a FES condition.

**Main outcome measures:**

We analyzed the joint kinematics (trunk, shoulder, and elbow) from the starting position until the affected hand reached the glass.

**Results:**

Participants receiving FES carried out the movement with less trunk flexion, while shoulder flexion elbow extension was increased, compared to placebo conditions.

**Conclusion:**

The application of FES to the UL and interscapular muscles of stroke patients with motor impairment of the UL has improved reaching movements.

## Introduction

Reaching movements in stroke patients are characterized by decreased amplitudes at the shoulder and elbow joints compared to healthy subjects (1–6). The movement pattern of patients with stroke is highly related to their level of motor function impairment, which becomes modified due to the lack of inter-articular coordination (1). There is a decrease in the range of motion at the elbow joint with a tendency toward flexion, which avoids correct extension of the upper limb (UL), hampering the ability to perform appropriate reaching movements. Excessive shoulder abduction is also observed as a compensatory movement when there is a lack of appropriate shoulder flexion (7).

In the case of the trunk, greater trunk displacements have been observed in patients with stroke, forward displacements, and torsion movements, which are related to deficits in elbow extension, and shoulder flexion and adduction, as compensatory mechanisms that occur during reaching movements or other activity. Patients are able to develop new motor strategies to achieve their goal despite UL deficits (1–7). There is a greater involvement of the trunk and scapula during the execution of reaching movements due to the creation of new movement strategies to compensate for the deficiencies (8).

The scientific literature has shown that stroke patients need to create new movement strategies. This involves the development of pathological synergies to carry out the desired movements. An example of this is the excessive movements of the trunk and scapula to compensate the deficiencies resulting from the pathology (7). Proper activation of the interscapular muscles depends on the position of the trunk. Stroke patients, due to the deficits affecting their trunk and scapular movement patterns, are under unfavorable conditions for being able to perform appropriate and selective activation of these muscles, which has a negative impact on the movement of the UL (9–11).

Regarding the UL muscles involved in reaching movements, a deficit in muscle control and activation has been observed (5, 12, 13). The synergistic contraction of the shoulder flexor and extensor muscles during reach becomes deteriorated due to muscle weakness and; therefore, the resulting movement is deficient (14). Furthermore, spastic muscle patterns may also prevent the correct performance of UL movements (15–18).

Functional electrical stimulation (FES) is a form of treatment that seeks to activate the paretic muscles using short-duration electrical pulses applied *via* surface electrodes through the skin (19). The use of FES and neuroprostheses has spanned almost four decades (20, 21). The use of FES as a neuroprosthesis consists of self-treatment at home by means of a neuroprosthetic neuromuscular stimulation system. The objective of this modality is to assist the performance of an activity of daily living (ADL) (22). Recently, functional and clinical improvements have been reported with the therapeutic application of FES, in which stimulation was used to increase voluntary movement after stroke (22, 23). Therapeutic FES modalities have been used to recruit UL muscles, improving weakness, the dyscoordination of single and multiple joints movements, and spasticity (24).

Most studies employing therapeutic FES for paretic UL rehabilitation are based on stimulation of the shoulder, elbow, and wrist muscles without recruitment of the interscapular muscles (25–28). The importance of an appropriate and specific contraction of the interscapular musculature during UL movement is necessary to adapt the position of the scapulothoracic joint to the degree of movement of the glenohumeral joint. This musculature has a stabilizing function upon the entire glenohumeral complex, which is necessary for a correct reaching movement (29–31). In healthy subjects, the posture of the trunk has been shown to influence changes in scapular movement and interscapular muscle activity during UL elevation (29, 32). The motor control of shoulder movement influences the correct and proper activation and synchronization of these muscles (33).

In this study, we tested the ability of a FES system to assist the UL movement of stroke patients based on the stimulation of interscapular, shoulder, elbow, wrist, and finger muscles. To our knowledge, no empirical study to date directly addresses this question. The authors hypothesized that participants receiving FES to the UL and interscapular muscles would be able to perform the movement with less trunk anteroposterior tilt and major shoulder flexion and elbow extension. The aim of this feasibility study was to evaluate whether the application of FES to the UL and interscapular muscles of stroke patients with UL motor impairment would be able to modify their reaching patterns, measured using instrumental movement analysis systems.

## Materials and Methods

### Subjects

A cross-sectional study was conducted. Recruitment was based on the voluntary participation of patients with stroke and UL motor function impairment. The participants were recruited from rehabilitation facilities and patient associations. Contact with prospective participants was made *via* meetings with their clinicians and informative flyers.

The selection procedure was made by non-probabilistic sampling of consecutive cases of patients who met the inclusion criteria: subjects older than 30 years and younger than 70 years, with a confirmed diagnosis of chronic stroke (more than 6 months of evolution), the ability to manipulate most objects; a modified Ashworth scale score ≤2 in the UL muscles, deltoid, triceps brachii, biceps brachii and to the wrist and finger flexor an extensor muscles, the ability to understand instructions and actively cooperate in the tasks indicated by a score ≥20 in the Mini-mental Test and suitable family and social support, to assist the patient in the use of the FES device at home. Patients with mixed aphasia, hemineglect, articular rigidities (irreducible contractures and arthrodesis), severe sensitivity alterations that impeded the use of the FES system, and skin conditions that could hamper or render impossible the application of the FES system were excluded. This protocol was approved by the local ethics committee of the Rey Juan Carlos University. Informed consent was obtained from all participants included in this study.

### Instrumentation

The Compex^®^ electrical stimulator was used for muscle stimulation. Developed in Zurich, this device is considered one of the most flexible and versatile FES systems available. It was designed to be used as a medical device for any FES application, either as a neuroprosthesis or as a research tool (34).

The VICON Motion System^®^ (Oxford Metrics, Oxford, UK) was used for the purpose of motion analysis. This system consists of eight 100 Hz infrared capture cameras and a data station where the information is gathered and processed through the VICON Upper Limb 2.0^®^ model (35).

### Procedure

The research took place at the Laboratory of Analysis of Movement, Biomechanics, Ergonomics, and Motor Control (LAMBECOM), located in the Department of Physiotherapy, Occupational Therapy, Rehabilitation, and Physical Medicine of the Faculty of Health Sciences of the Rey Juan Carlos University.

In the first place, all patients granted their consent to participate in this study by signing the informed consent. The study protocol consisted primarily of a muscle training program that the patients performed at their own homes for a week. The purpose of these sessions was for the patient to become accustomed to the sensation of electrical stimulation, as well as to the training of the UL muscles, in order to subsequently undergo the evaluation process satisfactorily.

The home training program consisted of two sessions per day, for 7 days, of 30 min of stimulation. In the first session, stimulation was applied to the wrist and finger extensor muscles and to the triceps brachii. In the second session, the anterior deltoid and interscapular muscles were stimulated. The training program was pre-defined and consisted of 2 min at 1 Hz, 200 µs with 7.5 s of stimulation, and 7.5 s of rest; 16 min at 25 Hz, 200 µs and 7.5 s of stimulation, and 7.5 s of rest; and 2 min at 1 Hz, 200 µs and 7.5 s of stimulation, and 7.5 s of rest. The participants were told to indicate all deleterious effects such as skin discomfort, referred pain, paresthesia, and uncomfortable muscle contractions.

Once the training period was carried out, the instrumental evaluation was performed in the LAMBECOM using the Vicon^®^ system. For this purpose, 12 passive reflective markers of 14 mm were placed in specific locations of the affected UL and trunk of the patients according to the Vicon UL model (35). The locations were: the spinous process of the seventh cervical vertebra, the spinous process of the 10th thoracic vertebra, the acromion, the center of the right scapular spine, the sternal manubrium, the xiphoid apophysis, the lateral epicondyle of the humerus, the middle third of the forearm, the radial styloid apophysis, the ulnar styloid apophysis, and the base of the third metacarpal bone.

**Figure 1:**
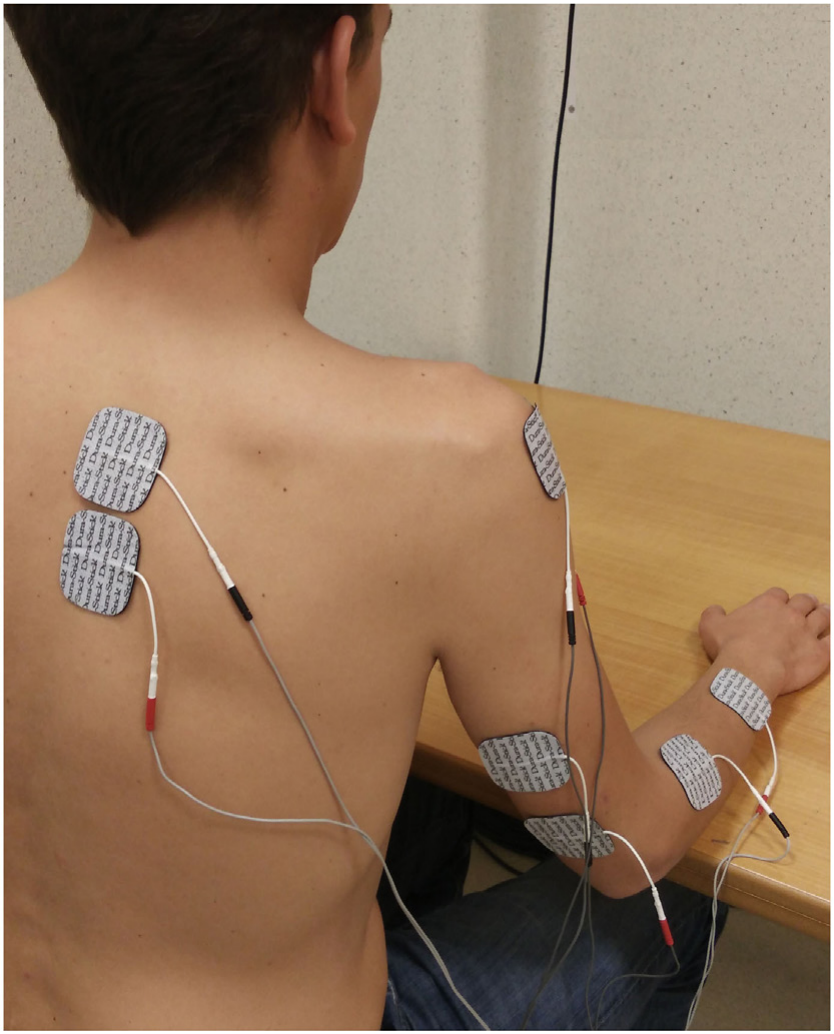
**Patient with the functional electrical stimulation device**.

Four muscle groups were selected for muscle stimulation: the interscapular muscles, the anterior deltoid, the triceps brachii, and the wrist and finger extensor muscles. Adhesive and square shaped Dura-Stick^®^ electrodes were used (5 cm × 5 cm) (Figure 1). The placement of these electrodes depended on the muscle area in which the best muscle contraction occurred. The frequency, pulse width, and duration of the FES onset ramp were set at 25 Hz, 200 µs, and 0.5 s, respectively, for the entire session. The current intensity was established according to the optimal amplitude necessary for producing the muscular contraction and the desired movement in each patient, and according to patient tolerance (submaximal contraction). Hence, prior to the evaluation, different trials were performed to choose the most appropriate intensity.

The kinematic analysis consisted of the patients performing six repetitions for each condition of the reaching task while being measured with a motion capture platform based on the Vicon Motion (Oxford Metrics, Oxford, UK) optoelectronic system.

The conditions were: condition 1: placebo; and condition 2: FES. In condition 1, the selected muscles were stimulated with electrical impulses, but with an amplitude that did not produce movement. In condition 2, electrical stimuli were applied to the triceps brachii, anterior deltoid, extensor muscles of the wrist, and fingers and interscapular muscles (lower trapezius and rhomboid), to favor the scapular approximation, the stabilization of the scapula with regard to the trunk, the external rotation of the shoulder, and the coaptation of the glenohumeral joint (31, 32).

To perform these trials, the patients were placed in a sitting position on a wooden chair without a backrest. The patient-to-desk distance was 8–10 cm. Patients were asked to put their hands on the desk (palms down) with their shoulder at around 15° flexion, 20° abduction, and with the elbow at around 90° flexion. A hard plastic glass (diameter = 5.5 cm, height = 15 cm) was used as the target. The glass was placed on the desk in line with the patient’s sternum and at a distance equal to 75% of the maximum reachable distance with the paretic arm.

Patients were instructed to reach and touch the glass from the starting position using their paretic hand and then they returned to the initial position. All patients practiced the reaching task before motion capture trials. Once this phase was completed, a static calibration recording was performed. Using this recording, we checked that each marker was visible from the scanning cameras and the analyzed movements were registered. In these, after the verbal instruction “Get ready…go,” patients had to lift their arm and reach and touch the glass at a comfortable speed.

For this, each patient performed the reaching task under two different conditions. Each condition was repeated six times in a randomized manner.

The sequence of muscular activation with electrical stimuli was: first the interscapular muscles to get the thorax to extend, followed by the anterior deltoid and the triceps brachii to achieve the reaching movement, and finally, the wrist and fingers extensor muscles to touch the glass.

### Outcome Measures

**Figure 2:**
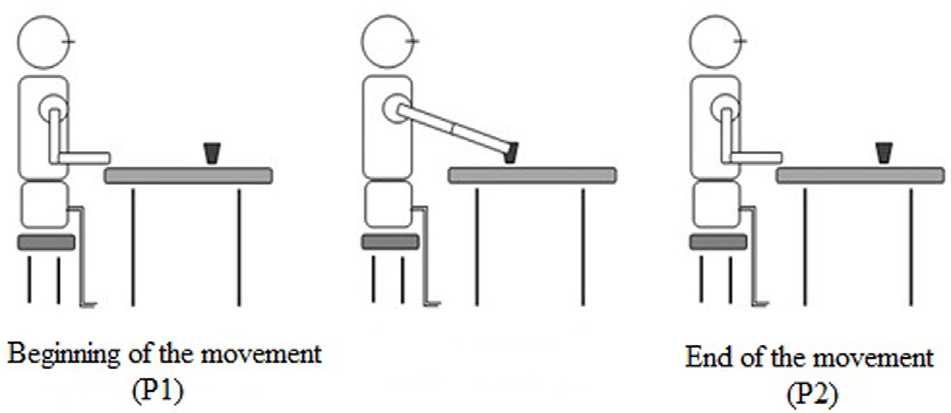
**Reaching movement**.

We analyzed the joint kinematics (trunk, shoulder, and elbow) when the affected hand reached the glass from the starting position. The end of the movement was considered as the point when the affected hand touched the glass. The beginning of the movement was when the trunk became straightened or extended because the stimulation with placebo and FES began (Figure 2).

We established the beginning of the movement using the Vicon Nexus software v1.8.5^®^, creating an event immediately when the stimulation begins and the verbal commands (“Get ready…go”). Another event was created at the end of the movement when the affected hand touched the glass and the stimulation ceased. Two raters evaluated these events, and if there were discrepancies between them, a third rater was consulted in order to reach an agreement.

The following kinematic parameters were analyzed: trunk, shoulder, and elbow joint angles at the beginning of the movement; trunk, shoulder, and elbow joint angles at the end of the movement; and trunk, shoulder, and elbow joint range of movement (ROM) during reaching. Range of motion was the difference between the joint angles (degrees) at the beginning and at the end of the reaching movement. Positive values in trunk, shoulder, and elbow joint angles indicated flexion. A negative value or a reduction in the value of the trunk, shoulder, and elbow positions indicated extension.

The Vicon Nexus software v1.8.5^®^ was used to calculate outcome measures based on the biomechanical model of the Vicon Upper limb^®^ model. The output angles for all joints were calculated from the YXZ cardan angles, derived by comparing the relative orientations of the two segments. The trunk angle was measured relative to the laboratory axes. The angle of the shoulder segment was relative to the proximal segment, i.e., the shoulder to the trunk. The angle of the elbow was a relative angle between the upper arm and the forearm (35).

Henmi et al. (36) evaluated the validity and reliability of the Vicon Upper Limb^®^ model in healthy subjects. For this, they examined the ranges of movement of the cervical spine, shoulder, elbow, and forearm, using the Vicon Motion System^®^ and a universal goniometer. The authors obtained an excellent Pearson correlation coefficient for shoulder flexion (0.94) and elbow flexion (0.91). In addition, the SD between the repeated measurements was very small: 0.78° for shoulder flexion and 0.89° for elbow flexion.

### Statistical Analysis

The statistical analysis was carried out using the SPSS statistical software system (SPSS Inc., Chicago, IL, USA; version 22.0). A normal distribution for the kinematic parameters was found using the Shapiro–Wilk test and Kolmogorov–Smirnov test.

The Student’s *t*-test for related samples was used for the analysis of kinematic parameters, comparing the data for the two conditions (FES and placebo). Descriptive statistics were used to summarize data, including calculation of the means and SDs for continuous data. The statistical analysis was performed with a confidence level of 95%, so that significant values were considered at *p* < 0.05.

## Results

The sample consisted of a total of 21 male patients with chronic stroke, of the 25 selected at the study onset. Four subjects were excluded because they were unable to attend the evaluation appointments due to logistical problems. The ages of the included participants ranged between 40 and 69 years old (with a mean age of 59.12 ± 10.31 years). The affected UL was analyzed in this study. Concretely, 11 patients presented left UL paresis (52.38%) and 10 has right UL paresis (47.62%). The affected UL was on the dominant side in 14 participants (66.67%) and, on the non-dominant side, in 7 participants (33.33%). The mean time since the stroke episode was 6.18 ± 3.12 years. Regarding the type of stroke, 38.10% (8) were ischemic and 61.90% (13) were hemorrhagic. The mean score of participants on the motor function domain of the UL-FMA was 26.87 out of 66 points (SD 10.6), with 72.25 out of 126 points (SD 20.41) on the total score of the UL-FMA (including the sensory, passive ROM, and pain subscales). The amplitude of the stimulation needed to produce the muscular contraction ranged between 17 and 42 mA, depending on the muscle groups and the stimulation threshold of each participant.

Table 1 summarizes the kinematic parameters studied for the trunk, shoulder, and elbow during the reaching task.

**Table 1:** **Joint kinematics in the sagittal plane (degrees)**.

	Placebo functional electrical stimulation (FES)	FES	Within subjects analysis		
			DM	CI 95%	*p*
Trunk P1	6.49 (2.09)	8.06 (2.21)	−1.57	From −3.14 to 0.005	0.054
Trunk P2	15.81 (5.73)	13.44 (5.38)	2.37	From 0.08 to 6.42	0.043*
Trunk ROM	7.31 (5.77)	5.37 (4.49)	−1.93	From −3.44 to −0.43	0.015*
Shoulder P1	15.69 (4.75)	15.71 (4.52)	−0.019	From −1.72 to 1.68	0.981
Shoulder P2	31.75 (16.25)	34.62 (15.31)	−2.86	From −4.43 to −1.30	0.001*
Shoulder ROM	16.05 (14.64)	18.90 (14.93)	−2.84	From −4.93 to −0.76	0.011*
Elbow P1	90.10 (5.51)	91.11 (2.73)	−1.01	From −3.37 to 3.98	0.998
Elbow P2	77.73 (14.71)	72.69 (16.44)	5.03	From 2.17 to 7.90	0.002*
Elbow ROM	−12.37 (11.74)	−17.40 (16.61)	5.03	From 0.42 to 9.64	0.034*

*ROM is range of movement (amplitude between the beginning and the end of the movement); a negative elbow range of motion indicates extension. P1 is the beginning of the reaching. P2 is the end of the reaching*.

*Data are expressed as mean (SD). DM is difference of means. CI 95% is confidence interval*.

*p-Value < 0.05 using Student’s t-test for related samples.

The participants with FES decreased their trunk ROM and showed less trunk flexion in the end of reaching compared to the participants with placebo FES. We did not observe significant differences in the angle of the trunk at the beginning; however, when the stimulation began, the participants with FES also began the reaching movement with less trunk forward flexion (i.e., from a more extended position).

During the reaching movement with FES, the participants increased the shoulder flexion at the end of the movement and the ROM compared to the participants who performed the reaching task with placebo FES.

The elbow joint showed significant differences in the final position when the affected hand reached the object. The participants increased their elbow extension using the FES compared to the placebo. In addition, the elbow ROM significantly increased in the FES condition compared to the placebo condition.

**Figure 3:**
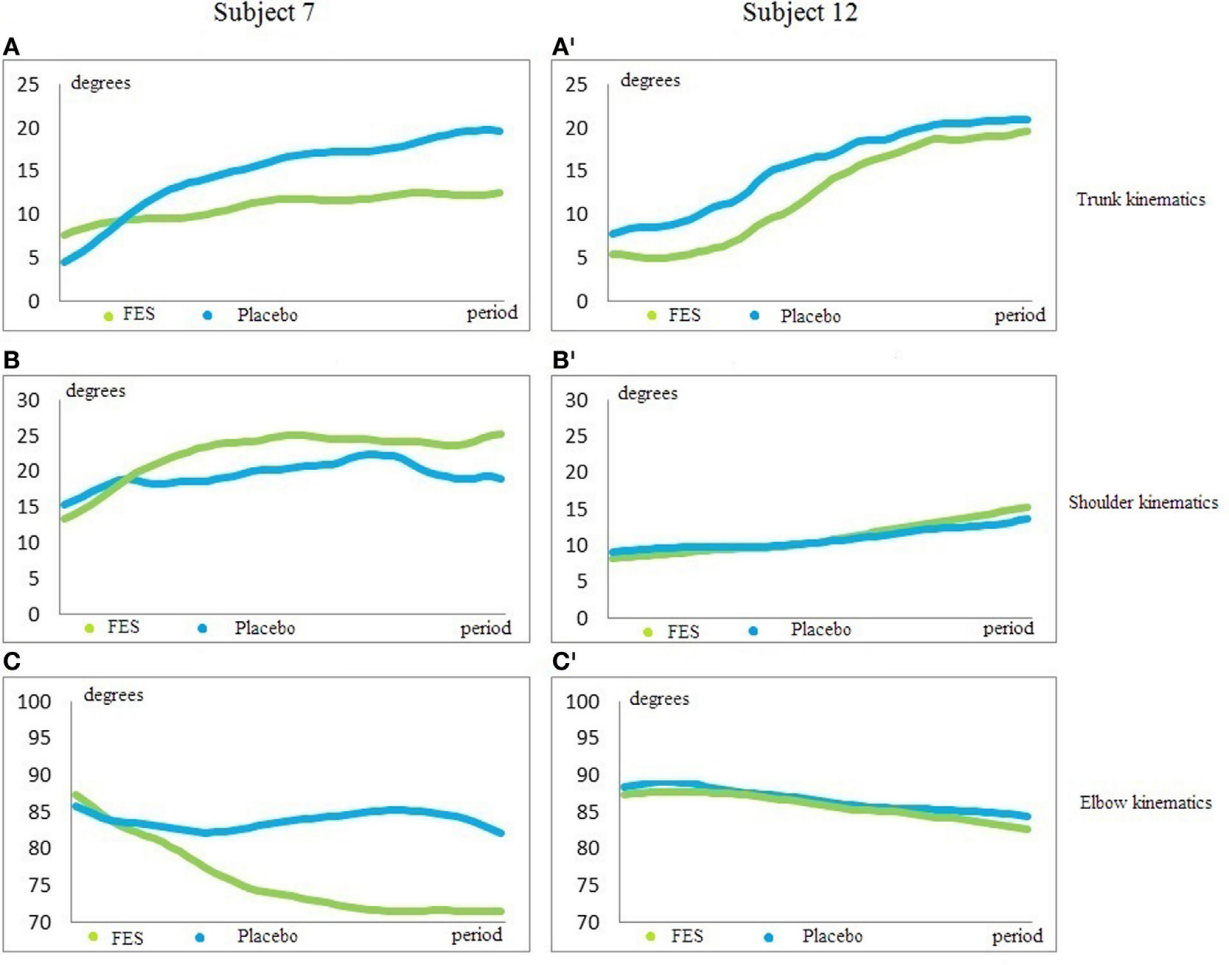
**Trunk, shoulder, and elbow kinematics**. **(A,A’)** is trunk kinematics; **(B,B’)** is shoulder kinematics; **(C,C’)** is elbow kinematics. *X*-axis shows degrees. *Y*-axis is the period between the beginning and the end of the motion. Blue line is the reaching pattern with placebo condition; green line is the reaching pattern with functional electrical stimulation (FES). The subjects begin the movement in a similar position in both conditions. However, the joint positions at the end of the movement are very different in each subject. The participant 7 with FES condition decreased the trunk flexion **(A)**, increased the shoulder flexion **(B)**, and increased the elbow extension **(C)**. The participant 12 with FES condition showed a smaller increase of shoulder flexion **(B’)** and elbow extension **(C’)** because the movement was performed with greater trunk flexion **(A’)**. Both participants performed an improved reaching movement with FES compared to placebo.

The trunk and arm position at the beginning of the movement was similar in both groups. In addition, the SD was low. However, when the stimulation began and the affected hand reached the glass, the SD increased in trunk, shoulder, and elbow P2 and ROM. This is probably because the participants use different patterns to perform the task. For example, some participants in the FES group showed a smaller increase of shoulder flexion and elbow extension because the movement was performed with greater trunk flexion (Figure 3).

## Discussion

The aim of this feasibility study was to evaluate whether the application of FES to the UL and interscapular muscles (rhomboid and lower trapezius), in stroke patients with motor impairment of the UL, would be able to modify patients’ reaching pattern, measured with a motion capture system.

The scientific literature available has shown that FES systems improve subjects’ reaching patterns. However, no studies have evaluated the effectiveness of the same using instrumental and objective motion capture systems. These systems help us to discriminate the UL pattern performed by each patient. Therefore, despite the heterogeneity of the sample, the use of the motion capture system has enabled us to examine each movement pattern in order to determine whether FES stimulation was able to modify the subjects’ UL pattern. To our knowledge, this is the first study using FES that specifically includes the interscapular muscles within the protocol of stimulated muscles.

Concerning the results obtained for the analyzed variables of the trunk movements in the sagittal plane, we have found significant differences in the trunk ROM and in the end position reached between the conditions. The trunk ROM during reaching was found to be lower in participants who performed the movement with FES, who also displayed a decrease in trunk tilt. This reduction in the ROM and trunk tilt may occur because the participants with FES began the movement with their trunk in a more neutral position. In addition, the interscapular stimulation may improve the trunk posture toward a more extend position. The motion of the scapula is influenced by muscle forces and joint reaction forces, which arise from the thoracic surface as well as the acromioclavicular and glenohumeral joints. Also, the stability of the scapula influences both the trunk and UL motions (37). It is probable that the initial stimulation of interscapular muscles may have helped these patients to achieve this posture. Different authors have shown that trunk flexion complicates the performance of UL tasks. For appropriate UL movements, a correct starting position at the level of the trunk and scapula is necessary (9, 10).

In the case of patients with stroke, the deficits that are typically present in their trunk and scapula movement patterns place them at a disadvantage for achieving an appropriate and selective activation of these muscles and, consequently, this has a detrimental effect on the movement of their UL (9, 10). Notably, a number of authors have reported that the excessive trunk movement of these patients is due to an inappropriate activation of the scapular musculature (8–10), which explains why stimulation at this level should improve the movement pattern with a lesser tendency toward forward trunk bending.

Regarding the application of electrostimulation to the muscles of the UL, the combination of proximal and distal stimulation may potentially restore function to a much larger group of patients compared to previous works that only stimulated hand opening (38, 39). In line with our findings, previous reports have shown that voluntary shoulder movement and reaching effort increases wrist and hand flexion force, requiring additional extension force to open the hand in stroke patients. The correct movement of the UL may reduce the grasp force and may be useful to assist the performance of ADLs in individuals with a hemiparetic arm (40). The importance of the stimulation of the triceps brachii has been demonstrated in previous studies, which have demonstrated that the torques generated by triceps stimulation for producing elbow extension may have beneficial effects during dynamic reaching (41).

In stroke subjects, the application of electrostimulation to the muscles of the UL has been found to enhance the reacquisition of motor skills with the affected UL and to increase the degree of shoulder flexion and elbow extension, thus assisting the reaching movement, as measured with motion analysis systems; our results confirm and extend these previous works (42–44) by demonstrating that FES applied to the UL muscles is effective for improving simple single joint movements as well as more complex reach-to-grasp movements performed with the hemiparetic UL. Makowski et al. (42) and Lew et al. (43) studied different reaching movements and ADLs in chronic stroke patients, *via* the application of FES. The results they obtained showed improvements in range distances, hand opening, an increase in forearm muscle activity, and the ability to complete ADLs in which shoulder flexion was required and in which elbow extension was required and which the patients were initially unable to perform. However, the authors of the aforementioned study did not report kinematic values for the different joints, which is why we are unable to discern which joint is able to explain the improvements in movement. Furthermore, the prior study did not stimulate the interscapular muscles and; therefore, we cannot compare our kinematic data. On the other hand, Koesler et al. (44) studied the kinematics of the UL while performing median nerve stimulation to the origin of the brachial biceps in 12 patients with chronic stroke. The results of their study demonstrated improvements in analytical UL movements and in complex movements, such as reach and grip. These data are comparable to our findings although in the case of the former study, there was no electrical stimulation at the muscle level, but rather, stimulation was performed directly upon the nerve. In contrast, after using FES and an exoskeleton for reaching and grasping in 18 patients with stroke, Grimm and Gharabaghi (45) stated that FES alone was insufficient for the correct performance of the selected movements in these patients. They concluded that the use of the relief device was essential to achieve the performance of the reach and grip exercises. Our results differ with the aforementioned study by suggesting that stroke patients are able to perform ROM with FES.

Recent research has shown that the repetitive practice of a motor task combined with positive feedback leads to the reorganization of the motor cortex. Changes in cortical excitability increase even more when motor tasks are performed, which require more skill on the part of the patient (46), thus the active participation in the performance of the task leads to a substantial increase in cortical excitability in comparison with passive or non-functional training (47). Somatosensory input is essential for motor learning, and it has been suggested that an increase in the excitability of corticospinal projections to the muscles of the paretic hand may facilitate functional recovery of dexterity after stroke (48). Corticospinal excitability can be increased by periods of electrical stimulation, transcranial direct current brain stimulation, or a combination of both (49).

In stroke rehabilitation, specific training or repetitive exercise can increase corticospinal excitability and improve function of the paretic hand (50). Therefore, one of the main advantages of FES is the ability of the patient to perform repetitive movements in any environment, which significantly enhances their ability to recover (51). In addition to enhancing function, FES enables people to engage in therapies that require a prescribed level of voluntary ability. Thus, the use of FES during and after therapy is most likely of value in cases of moderate to severe chronic deficiency, where therapeutic interventions to date have been less effective (26). The literature suggests that a small number of individuals with severe impairments are unable to regain motor control in the UL. These individuals with chronic stroke suffer from weakness, spasticity, atrophy, and stiff joints due to the stroke and learned non-use. Our findings suggest that the use of FES may be beneficial for such individuals. We have shown that FES can produce functional arm movement and is able to reduce the pathological pattern of the reaching movement in these patients, such as excessive trunk bending instead of a greater shoulder flexion and elbow extension.

According to the results of this study, the stimulation of interscapular and UL muscles using FES increases the range of motion of the shoulder and elbow compared to placebo conditions. Most studies have used FES stimulation for UL muscles without stimulating the interscapular muscles. These studies reported that the UL ROM also increased. Therefore, further studies are required comparing which type of stimulation is most appropriate: FES for UL muscles alone or a combined stimulation with FES for UL and interscapular muscles. In addition, it is important for future works to include kinematic data of the UL joint in order to compare our results regarding kinematics with other types of FES stimulation. The study demonstrates the suitability of the proposed stimulation with FES, because there were not deleterious events during the application, the performance of the intervention was easy and the systems used for stimulation are accessible. Therefore, it would be possible to carry out this type of intervention in clinical environments.

The present study has several limitations that need to be addressed in the future. Due to the clinical variability inherent to stroke, it was difficult to form a homogeneous experimental group in terms of motor characteristics such as movement patterns. However, despite the heterogeneity of the sample, significant changes in joint kinematics have been observed. Another limitation is that the same patient group was treated under two different conditions. Future studies should include independent groups and add a follow-up to understand the effects of FES stimulation over time. Also, the interscapular muscle stimulation with percutaneous FES was unable to specifically stimulate the rhomboid muscle or lower fibers of trapezius. It is possible that a more specific stimulation of these muscles may improve the trunk position and generate a more physiological reaching movement. In addition, further works should analyze the ROM of trunk movement from the start of the stimulation to the start of the reaching in order to evaluate the influence of the stimulation on the interscapular muscles in trunk posture. Finally, this study did not analyze the wrist and finger kinematics due to the fact that the motion capture systems are shown to be more reliable for shoulder and elbow movements (36).

## Conclusion

We provide kinematic evidence that the application of FES in the UL and interscapular muscles of stroke patients with motor impairment of the UL has reduced the trunk tilt and increased the shoulder flexion and elbow extension, improving the reaching movement, compared to the placebo stimulation. In addition, the stimulation of interscapular muscles (rhomboid and lower trapezius) may help to improve the trunk position during the UL movements through the scapula. To our knowledge, this is the first study to use a protocol that includes the stimulation of these muscles. The findings of this feasibility study show that FES has no side effects and that the electrical dose described in this manuscript could be taken into account in a large study. Further trials with follow-up, a control group, and larger samples are necessary to compare the effectiveness of this modality of FES.

## Ethics Statement

The study was approved by the Human Ethics Committee of the Rey Juan Carlos University.

## Author Contributions

All the authors have contributed equally to the research.

## Conflict of Interest Statement

The authors declare that the research was conducted in the absence of any commercial or financial relationships that could be construed as a potential conflict of interest.

## Abbreviations

ADL, activities of daily living; FES, functional electrical stimulation; LAMBECOM, laboratory of analysis of movement, biomechanics, ergonomics, and motor control; UL, upper limb; ROM, range of movement.
